# The heart cannot pump blood that it does not receive

**DOI:** 10.3389/fphys.2014.00360

**Published:** 2014-09-18

**Authors:** Wouter Wieling, Frederik J. de Lange, David L. Jardine

**Affiliations:** ^1^Department of Internal Medicine, Academic Medical Centre, University of AmsterdamAmsterdam, Netherlands; ^2^Department of Cardiology, Academic Medical Centre, University of AmsterdamAmsterdam, Netherlands; ^3^Department of General Medicine, Christchurch Hospital, University of OtagoChristchurch, New Zealand

**Keywords:** gravitational stress, orthostasis, heart rate, cardiac output, vasoconstriction, orthostatic tolerance

Orthostatic gravitational stress results in a decrease in venous return. Because the heart cannot pump blood that it does not receive, ventricular stroke volume and cardiac output decline, but until the point of presyncope, there is little change in arterial pressure. There is little doubt that the vasomotor outflow to the resistance vessels and presumably the splanchnic capacitance vessels are of fundamental importance in the prevention of hypotension (Rowell, 1993; Wieling and van Lieshout, 2008), but there is also a concomitant increase in heart rate and the significance of this is much less clear (Hainsworth, 2000).

In a recent issue of Frontiers in Physiology, Convertino focussed on the physiology of maximal compensation to orthostasis studying high and low tolerant healthy subjects. Exposure to a maximal lower body negative pressure (LBNP) test was used to quantify orthostatic tolerance. The author argues that this stressor is equivalent to actual orthostasis. However, recent studies suggest that pooling in the splanchnic area during actual orthostasis (standing/head-up tilting) is more important than previously reported in studies using simulated orthostasis by applying lower-body negative pressure up to the levels of the iliac crest (Taneja et al., 2007). More pronounced splanchnic pooling during orthostasis may result in more stimulation of vascular subdiaphramatic receptors that are postulated to play a role in orthostatic adjustment by causing vasoconstriction and augmentation of the carotid baroreflex (Doe et al., 1996). Other obvious differences with LBNP are that during free standing the carotid baroreceptors are always above the heart and the static increase in skeletal muscle tone during active standing opposes pooling of blood in limb veins (Wieling and van Lieshout, 2008; Wieling et al., 2014). Increases in skeletal muscle tone are a key factor in orthostatic adjustment. Accordingly, a static increase in skeletal muscle tone by leg-crossing during a maximal LBNP test increases time to presyncope considerably (Krediet et al., 2006). Therefore, a device which combines head-up tilting with negative pressure to the lower part of the body (Hainsworth, 2000) seems a more physiological approach to quantify orthostatic tolerance in a reproducible way. Although LBNP (in the horizontal position) is a deficient model for studying orthostasis, it can be used to simulate loss of central blood volume (hemorrhage) (Johnson et al., 2014).

As far as the physiology of maximal hemodynamic compensation in high and low tolerant healthy subjects, the author reaffirms the concept that the heart rate response contributes significantly to orthostatic tolerance (Convertino, 2014). The observations of Convertino are of considerable interest, but it important to realize that they are based on studies in young adult healthy subjects exposed to a maximal LBNP stress. The observations cannot be generalized to the adjustment to the upright posture during free standing. Based on clinical observations the following data indicate that neural heart rate control is not important for orthostatic tolerance.

1. Weissler studied the effects of posture and atropine on the cardiac output in six young adult male subjects. He documented that atropine administered in the supine position increased heart rate on average by 44 beats/min. The heart rate increase was accompanied by a cardiac output rise of about a factor 2 with an increase in mean arterial pressure. After administration of atropine in the upright posture heart rate increase by 65 beats/min, but no effect on cardiac output and blood posture were observed. When pooling of blood in the upright posture was prevented by sustained inflation of an anti-gravity suite, the cardiac responsiveness to atropine in tilted subjects was restored, in part (Weissler et al., 1957a). In another study it was documented that administration of atropine could not prevent an impending vasovagal faint (Weissler et al., 1957b).
2. Patients with a cardiac transplants have no increase in heart rate on standing, but intact orthostatic blood pressure control (Figure 1, left panel) (Van Lieshout et al., 1989; Wieling and Karemaker, 2013).
3. In patients with sympathetic vasomotor lesions, but intact vagal heart rate control pronounced orthostatic hypotension occurs despite an impressive postural tachycardia (Figure 1, right panel) (Wieling and Karemaker, 2013).
4. Atrial tachypacing at best has marginal effects on hypotension in patients with severe orthostatic hypotension due to autonomic failure (Sahul et al., 2004) and cardiac pacing does not improve orthostatic tolerance in patients with vasovagal syncope (El-Bedawi et al., 1994). However, benefit from rate-drop pacing in older patients with documented prolonged asystolic syncope has been reported (Brignole et al., 2012).
5. Another example of the disconnect between the heart rate responses and orthostatic tolerance are patients with the postural orthostatic tachycardia syndrome in whom very high orthostatic heart rates are associated with orthostatic presyncope. The treatment in these patients is aimed at decreasing postural complaints by *decreasing* the postural tachycardia (Joyner, 2011).

**Figure 1:**
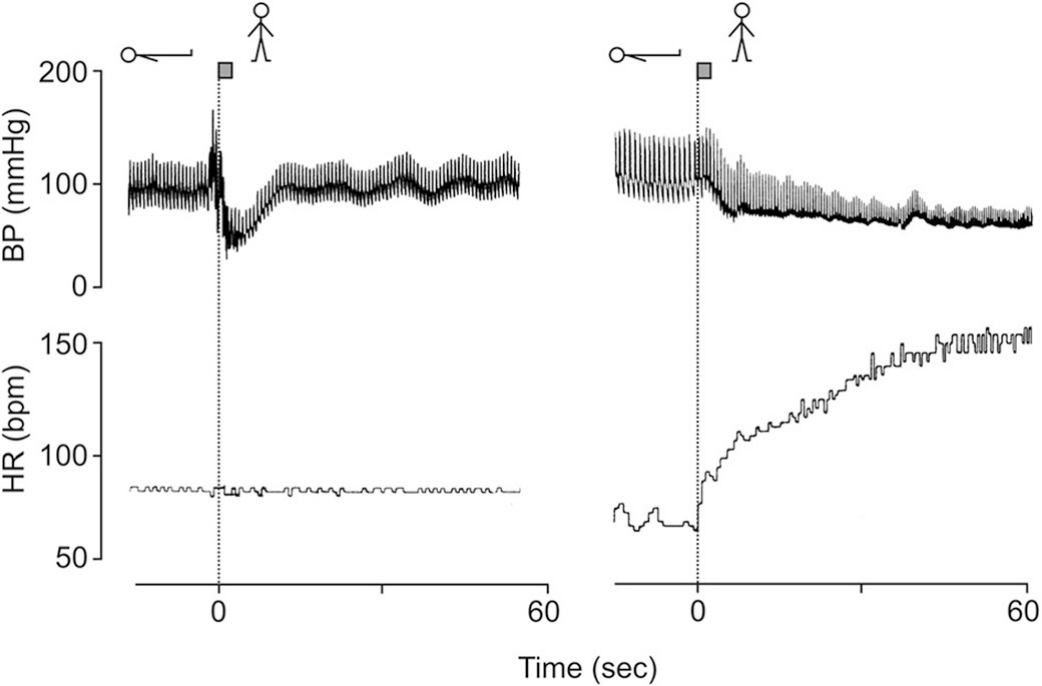
**Blood pressure and heart rate responses induced by active standing in a 38 year-old fit patient with a cardiac transplant (left panel) and a 23-year-old female patient with orthostatic hypotension (hypoadrenergic) with intact heart rate control (right panel)**. Revised after Wieling and Karemaker (2013) with permission of Oxford University press.

These data strongly support the view that the central venous reservoir is an important determinant of cardiac responsiveness to changes in heart rate i.e., a heart cannot pump blood that it does not receive. In fact, very high heart rates would decrease cardiac filling time and during conditions of impaired venous return such as orthostatic stress, could actually impair cardiac output (Hainsworth, 2000).

The positive correlation between orthostatic tolerance and the maximal sympathetically mediated heart rate before syncope in high and low tolerant healthy subjects observed by Convertino may have been because in this situation heart rate is a marker for enhanced sympathetic drive to resistance vessels, which provide the main defense against hypotension (Rowell, 1993; Hainsworth, 2000).

In conclusion, The arterial (and especially carotid) baroreceptor control of sympathetic motor tone of resistance and splanchnic capacitance vessels in combination with the central blood volume are the most important components in the maintenance of postural normotension in humans (Rowell, 1993; Wieling and van Lieshout, 2008). Activation of the skeletal muscle pump in the lower body can compensate in part for defects in control of vasomotor tone and a reduction of central blood volume. Cardiac effector mechanisms appear not to be important for the adjustment of arterial pressure to the upright posture.

## Conflict of interest statement

The authors declare that the research was conducted in the absence of any commercial or financial relationships that could be construed as a potential conflict of interest.

## References

[B1] Brignole M. Menozzi C. Moya A. Andresen D. Blanc J. J. Krahn A. D. (2012). Pacemaker therapy in patients with neurally-mediated syncope and documented asystole. Third international Study on syncope of Uncertain etiology (ISSUE-3). Circulation 125, 10–16 10.1161/CIRCULATIONAHA.111.082313 22565936

[B2] Convertino V. A. (2014). Neurohumoral mechanisms associated with orthostasis: reaffirmation of the significant contribution of the heart rate response. Front. Physiol. 5:236 10.3389/fphys.2014.00236 25071585 PMC4074989

[B3] Doe C. P. A. Drinkhill M. J. Myers D. S. Self D. A. Hainsworth R. (1996). Reflex vascular responses to abdominal venous distension in the anesthesized dog. Am. J. Physiol. 271(3 Pt 2), H1049–H1056 8853340 10.1152/ajpheart.1996.271.3.H1049

[B4] El-Bedawi K. M. Wahbha M. M. A. E. Hainsworth R. (1994). Cardiac pacing does not improve orthostatic tolerance in patients with vasovagal syncope. Clin. Auton. Res. 4, 233–237 10.1007/BF01827427 7888741

[B5] Hainsworth R. (2000). Heart rate and orthostatic stress. Clin. Auton. Res. 10, 323–325 10.1007/BF02322255 11324987

[B6] Joyner M. J. (2011). Exercise training in postural orthostatic tachycardia syndrome: blocking the urge to block beta-receptors. Hypertension 58, 136–137 10.1161/HYPERTENSIONAHA.111.173872 21690485

[B7] Johnson B. D. van Helmond N. Curry T. B. van Buskirk C. M. Convertino V. A. Joyner M. J. (2014). Reductions in central venous pressure by lower body negative pressure or blood loss elicit similar hemodynamic responses. J. Appl. Physiol. 117, 131–141 10.1152/japplphysiol.00070.2014 24876357 PMC4459917

[B8] Krediet C. T. van Lieshout J. J. Bogert L. W. Immink R. V. Kim Y. S. D. Wieling W. (2006). Legcrossing improves orthostatic tolerance in healthy subjects: a placebo-controlled crossover study. Am. J. Physiol. Heart Circ. Physiol. 291, H1768–H1772 10.1152/ajpheart.00287.2006 16714361

[B9] Sahul Z. H. Trusty J. M. Erickson M. Low P. A. Shen W. (2004). Pacing does not improve hypotension in patients with severe orthostatic hypotension. Clin. Auton. Res. 14, 255–258 10.1007/s10286-004-0202-2 15316843

[B10] Rowell L. B. (1993). Human Cardiovascular Control, 1st Edn. Oxford: Oxford University Press

[B11] Taneja I. Moran C. Medow M. S. Glover J. L. Montgomery L. D. Stewart M. (2007). Differential effects of lower body negative pressure and upright tilt on splanchnic blood volume. Am. J. Physiol. Heart Circ. Physiol. 292, H420–H426 10.1152/ajpheart.01096.2006 17085534 PMC4517828

[B12] Van Lieshout J. J. Wieling W. Wesseling K. H. Karemaker J. M. (1989). Pitfalls in the assessment of cardiovascular reflexes in patients with sympathetic failure but intact vagal control. Clin. Sci. 76, 523–528 2721119 10.1042/cs0760523

[B13] Weissler A. M. Leonard J. J. Warren J. V. (1957a). Effects of posture and atropine on he cardiac output. J. Clin. Invest. 36, 1656–1662 10.1172/JCI103566 13491696 PMC1072777

[B14] Weissler A. M. Warren J. V. Estes E. H. McIntosh H. D. Leonard J. J. (1957b). Vasodepressor syncope; factors influencing cardiac output. Circulation 15, 875–882 10.1161/01.CIR.15.6.875 13437412

[B15] Wieling W. van Lieshout J. J. (2008). Maintenance of postural normotension in humans, in Clinical Autonomic Disorders: Evaluation and Management, ed Low P. A. (Boston, MA: Little, Brown and Company), 57–67

[B16] Wieling W. Karemaker J. M. (2013). Recording of heart rate and blood pressure in the evaluation of neuro-cardiovascular control, in Autonomic Failure. A Textbook of Clinical Disorders of the Autonomic Nervous System, 4th Edn., eds Mathias C. J. Bannister R. (Oxford: Oxford University Press), 290–306 10.1093/med/9780198566342.003.0025

[B17] Wieling W. van Dijk N. Thijs R. D. de Lange F. J. Krediet C. T. P. Halliwill J. R. (2014). Physical countermeasures to increase orthostatic tolerance. J. Intern. Med. [Epub ahead of print]. 10.1111/joim.12249 24697914

